# Do androgen deprivation and the biologically equivalent dose matter in low‐dose‐rate brachytherapy for intermediate‐risk prostate cancer?

**DOI:** 10.1002/cam4.820

**Published:** 2016-07-25

**Authors:** Ryuji Tabata, Takahiro Kimura, Hidetoshi Kuruma, Hiroshi Sasaki, Masahito Kido, Kenta Miki, Hiroyuki Takahashi, Manabu Aoki, Shin Egawa

**Affiliations:** ^1^ Department of UrologyJikei University School of MedicineTokyoJapan; ^2^ Department of PathologyJikei University School of MedicineTokyoJapan; ^3^ Department of RadiologyJikei University School of MedicineTokyoJapan

**Keywords:** Androgen deprivation therapy, brachytherapy, dose–response, prostate cancer

## Abstract

The objective of this study was to investigate the impact of the biologically equivalent dose (BED) on treatment outcomes after iodine‐125 low‐dose‐rate brachytherapy (LDR‐BT) with or without supplemental external beam radiotherapy (EBRT) and androgen deprivation therapy (ADT) for intermediate‐risk prostate cancer (PCa). We retrospectively evaluated 292 Japanese patients. The impact of the BED and ADT on treatment outcomes was investigated. Cox proportional hazard models were used for univariate and multivariate analysis with biological progression‐free survival (bPFS) and clinical progression‐free survival (cPFS) as the primary outcome measures. The median follow‐up was 66 months. The bPFS and cPFS rates at 5‐/7‐years were 91.6/87.7% and 95.9/94.0%, respectively. When stratified by BED levels, the bPFS rates at 5‐/7‐years were 92.1/89.3% for <178.0 Gy_2,_ and 91.2/86.0% for ≥178.0 Gy_2_, respectively (*P *> 0.05). Based on ADT duration, the bPFS rates at 5‐/7‐years were 89.8/83.5%, 89.7/89.7%, and 97.5/97.5% for none, 1–3 months, and 4–12 months, respectively (*P* = 0.03). For the univariate analysis, the use of ADT and its duration were significant predictors for bPFS, whereas BED was not significant. A multivariate analysis did not indicate the use of ADT itself was significant, however, when covariates were accounted for by the duration of ADT, the longer use of ADT was found to significantly improve bPFS. Although cPFS was associated neither with the BED levels nor ADT duration (*P* > 0.05), ADT duration had a trend of improving cPFS (*P *= 0.053). The higher levels of BED did not significantly impact bPFS for intermediate‐risk PCa after LDR‐BT with or without supplemental EBRT and ADT. The longer duration of ADT could provide an additional benefit in the context of high‐dose irradiation generated by LDR‐BT.

## Introduction

The two most important advances in the contemporary radiotherapeutic management of localized prostate cancer (PCa) have centered on dose escalation and the use of androgen deprivation therapy (ADT). The value of adjuvant ADT with external beam radiotherapy (EBRT) for high‐risk PCa is well established 1, 2. Nevertheless, these trials used relatively low radiation doses that are suboptimal based on current standards. Iodine‐125 low‐dose‐rate brachytherapy (LDR‐BT) is a well‐established treatment for localized PCa. Its advantage stems from the highly conformal nature of the procedure and the known delivery of a higher radiation dose than EBRT. However, the ability of ADT to maintain its synergistic effect in this setting of high‐dose irradiation remains unknown. In a recent retrospective report by Stock et al. that included all‐risk groups of PCa, ADT improved biological progression free‐survival (bPFS) after LDR‐BT only at lower biologically equivalent dose (BED) levels, suggesting a less supplemental role in more heavily irradiated glands 3. To investigate the role and optimal duration of ADT use in high‐dose irradiation, there are several randomized trials ongoing (GETUG 14, EORTC 22991, RTOG 08‐15 and DART01/05 GICOR), of which DART01/05 GICOR showed, in terms of overall survival (OS), long‐term ADT (28 months) to be superior to short‐term ADT (4 months) with only high‐risk disease, but not with intermediate‐risk patients 4, 5, 6, 7. No randomized trials investigating the impact of ADT in conjunction with LDR‐BT have been published, therefore, we currently rely on retrospective series to guide our management 8. Thus, we aimed to investigate the influence of BED levels and its possible interaction with ADT in LDR‐BT with or without supplemental EBRT for intermediate‐risk PCa.

## Materials and Methods

### Patients

We retrospectively evaluated a total of 301 consecutive patients with intermediate‐risk PCa treated with LDR‐BT with or without supplemental EBRT at Jikei University Hospital from October 2003 to December 2009. Nine patients were excluded from the study because of a lack of BED data in one patient and a prolonged period of ADT >13 months in eight patients. Clinical stage was assigned based on the results of digital rectal examination, computed tomography (CT) and/or magnetic resonance imaging (MRI), and bone scintigraphy. The clinical stages ranged from T1c to T2bN0M0 as defined under the unified TNM system 9. Tumor grade was determined according to the Gleason grading system. Intermediate‐risk patients were defined according to D'Amico's risk groups for patients with one or more of the following disease characteristics: GS 7, clinical stage T2b, and prostate‐specific antigen (PSA) value 10.1–20 ng/mL 10. A single pathologist (H.T.) reviewed all prostatic biopsy specimen slides. The proportion of involved biopsy cores was calculated by dividing the number of evaluated cores and categorized as <50% or ≥50% (percent positive biopsy core rate; PPC). Interval follow‐up was started at the date of implantation of LDR‐BT. Follow‐up was censored at the last PSA recorded. The Jikei University Ethics Committee Institutional Review Broad approved this study. Patients provided written informed consent before treatment.

### Radiation treatment

All patients were treated with an ultrasound‐guided technique using the Mick applicator as previously described 11. We started with preplanning technique at the introduction of LDR‐BT and later adopted intraoperative planning 12. Iodine‐125 seeds were used in all patients. The prescribed doses of LDR‐BT were 145 Gy for monotherapy and 110 Gy in combination with pelvic EBRT. Supplemental EBRT was delivered using 3‐D conformal or intensity‐modulated radiation therapy techniques. The total average EBRT dose was 40–46 Gy (median, 45 Gy), and a daily fraction of 2.0 Gy was administered 5 days per week for 5 weeks. The indication of supplemental EBRT was decided at the discretion of treating physicians.

### Androgen deprivation therapy

ADT was administered to 150 (51.4%) patients before and during LDR‐BT and continued for up to 12 months. A 1‐ or 3‐month depot injection of LHRH agonist and bicalutamide (80 mg/day) was used for combined blockade in neoadjuvant ADT. LHRH agonists were used as adjuvant therapy during and after irradiation. Thirty‐four patients received 9 months of adjuvant ADT after LDR‐BT according to the protocol of the multi‐institutional, phase III, randomized controlled trial for intermediate‐risk PCa (SHIP0804) 11.

### Follow‐up

All patients underwent a CT scan for post‐implant dosimetry 1 month after LDR‐BT. The V100 index was measured as the percent of the target volume covered by the prescription dose. The dose–‐volume histogram provided the dose delivered to 90% of the prostate (D90). We used BED calculations with an *α*/*β* ratio of 2 Gy to compare the total radiation dose, consistent with the study by Stock et al 13. Biochemical recurrence (BCR) was defined as a PSA >2 ng/mL above the nadir, using the Phoenix definition 14. Local progression (LP) was defined as the reappearance of a biopsy‐proven local tumor at the primary site and/or the local tumor recurrence detected by MRI. When LP was detected by both biopsy and MRI, the prostate biopsy was given priority over MRI in the date of recurrence. Multiple imaging studies, including chest X‐ray, CT scan, bone scintigraphy and MRI, were used for patients with BCR to determine distant failure (DF). Patients enrolled in SHIP0804 were offered prostatic biopsies 36 months after LDR‐BT irrespective of the PSA values, as required by the protocol of the SHIP36B study. Other patients who developed BCR also underwent a prostatic biopsy. Toxicity was reported according to the National Cancer Institute Common Toxicity Criteria for Adverse Events, Version 4.0 (CTCAE ver.4.0).

### Statistics

Categorical and continuous variables were compared using the chi‐square and Kruskal–Wallis test. A Kaplan–Meier analysis and the log‐rank test were used to determine bPFS, clinical progression‐free survival (cPFS), and OS. cPFS was defined as the survival without documented clinical progression (CP), that is, LP and/or DF. Cancer‐specific mortality was estimated using the cumulative incidence method. The impact of the patient, tumor, and treatment characteristics on the bPFS and cPFS was examined using univariate analysis. Variables with *P* < 0.05 were entered into a forward conditional multivariate Cox proportional hazard regression model. For all analyses, a *P* < 0.05 was considered to indicate significant differences. All analyses were performed with the EZR, which is a graphical user interface for R (The R Foundation for Statistical Computing, Vienna, Austria) 15.

## Results

### Patient characteristics

The demographics of the 292 patients are shown in Table 1. The median age of the patients was 69 years (range: 51–81). Patients were treated with LDR‐BT alone (*n *= 234, 80.1%) or LDR‐BT combined with EBRT (*n *= 58, 19.9%). The median follow‐up was 66 months (range: 5–122) for all patients. The median BED level was 178.0 Gy_2_ (range: 77.9–229.1 Gy_2_): the number of patients with BED more than 200 Gy_2_ was 43 (14.7%). To explore the influence of BED levels, patients were stratified into low‐dose (<178.0 Gy_2_) and high‐dose (≥178.0 Gy_2_) groups by the median BED value.

**Table 1:** Treatment characteristics of 292 patients with LDR‐BT

Characteristics	No.	%	Mean	Median	SD	Range
Age (years)						
Total	292	100.0	68.8	69.0	5.5	51–81
<70	152	52.1	64.5	65.0	3.6	–
≥70	140	47.9	73.5	73.0	2.5	–
Follow‐up (month)						
Total	292	–	69.7	66.0	24.1	5–122
BED (Gy2)						
Total	292	100.0	177.9	178.0	21.5	72.9–229.1
<178.0	146	50.0	161.2	165.0	15.9	72.9–177.9
≥178.0	146	50.0	194.2	191.5	11.1	178.1–229.1
PSA (ng/mL)						
Total	292	100.0	9.0	8.4	3.7	2.7–19.1
<10	193	66.1	6.7	6.5	1.7	–
10–20	99	33.9	13.2	12.7	2.4	–
GS						
6	33	11.3	–	–	–	–
7	259	88.7	–	–	–	–
Primary Gleason grade						
3	213	72.9	–	–	–	–
4	79	27.1	–	–	–	–
T stage						
T1	233	79.8	–	–	–	–
T2	59	20.2	–	–	–	–
PPC						
<50%	205	70.2	–	–	–	–
≥50%	87	29.8	–	–	–	–
ADT use						
No	142	48.6	–	–	–	–
Yes	150	51.4	5.9	4.0	0.3	1–12
For 1–3 month	70	24.0	2.5	3.0	0.8	*–*
For 4–12 month	80	27.4	8.9	9.0	3.1	*–*
EBRT						
Yes	58	19.9	–	–	–	–
No	234	80.1	–	–	–	–
V100 (%)						
Total	292	100.0	94.6	95.8	6.1	23.3–99.9
With EBRT	58	19.9	94.8	95.5	4.5	73.2–99.9
Without EBRT	234	80.1	94.6	95.8	6.4	23.3–99.9
D90 (Gy)						
Total	292	100.0	159.9	162.9	23.8	71.1–202.8
With EBRT	58	19.9	131.6	132.0	16.5	91.3–173.7
Without EBRT	234	80.1	166.9	167.6	19.8	71.1–202.8

LDR‐BT, Low‐dose‐rate brachytherapy; BED, biologically equivalent dose; PSA, prostate‐specific antigen; GS, Gleason score; PPC, percent positive biopsy core rate; ADT, androgen deprivation therapy; EBRT, external beam radiotherapy; SD, standard deviation.

As for ADT, 142 patients were treated by radiotherapy alone (48.6%), and 150 patients by radiotherapy with ADT (51.4%). For statistical analysis, patients with ADT were stratified into subgroups (ADT use for none, 1–3 vs. 4–12 months). This categorical cutoff of 3 months was arbitrarily chosen, however, it is a part of our common practice and adopted as a protocol therapy in SHIP0804 multi‐institutional randomized controlled study 11. Seventy patients were treated with ADT for 1–3 months (short‐ADT group, 24.0%), and 80 patients for 4–12 months (long‐ADT group, 27.4%). The median durations of ADT in the short‐ and long‐groups were 3 and 9 months.

### Outcomes

As listed in Table 2, the BCR rate for all patients was 10.3% (30/292). The median time to BCR was 34.5 months (range: 9–98 months). Each BCR rate for the 2 groups stratified by BED levels was as follows: 8.9% (13/146) for the low‐dose group, and 11.6% (17/146) for the high‐dose group (*P > *0.05; Tables 2, 3). For each BED group, the median time to BCR was 36 months for low dose and 33 months for high dose (*P *> 0.05). The BCR rates for the no, short‐ and long‐ADT groups were 14.8% (21/142), 10.0% (7/70), and 2.5% (2/80), respectively (*P* value shown in Table 3). The median time to BCR for each ADT group was 57 (no), 21 (short‐ADT), and 17.5 (long‐ADT) months, respectively (*P *< 0.01). CP was associated neither with the BED levels nor ADT duration (*P* > 0.05, Table 3). The occurrence of BCR and CP is stratified by BED and duration of ADT in Table 4. In the low‐dose group, the longer use of ADT was related to less incidence of BCR (HR* *= 0.36 [95% CI = 0.14–0.93] *P *= 0.04) and CP (*P *= 0.02), however, in high‐dose group, no significant relationship was found between these groups.

**Table 2:** BCR and CP

	No.	(%)	ADT duration (Month)			BED level	
			0	1–3	4–12	<178.0	≥178.0
Total no.	292	(100.0)	142	70	80	146	146
BCR	30	(10.3)	21	7	2	13	17
CP (without duplication)	13	(4.5)	10	3	0	7	6
Site of CP (with duplication)							
Local progression	5	(1.7)	3	2	0	1	4
Lymph node involvement	3	(1.0)	2	1	0	2	1
Bone metastasis (died)	4	(1.4)	4 (1)	0	0	3	1 (1)
Lung metastasis	3	(1.0)	3	0	0	3	0

BCR, biochemical recurrence; CP, clinical progression; ADT, androgen deprivation therapy; BED, biologically equivalent dose.

**Table 3:** Cox proportional HRs of BCR and CP

	BCR			CP		
	*P* value	HR	95% CI	*P* value	HR	95% CI
BED						
<178.0	–	1.0	Reference	–	1.0	Reference
≥178.0	>0.05	1.43	0.69–2.96	>0.05	0.89	0.30–2.67
ADT						
No use	–	1.0	Reference	–	1.0	Reference
1–3 months	>0.05	0.81	0.34–1.91	>0.05	0.76	0.21–2.77
4–12 months	0.02	0.18	0.04–0.76	>0.05	N/A	N/A

HR, hazard ratio; BCR, biochemical recurrence; CP, clinical progression; BED, biologically equivalent dose; ADT, androgen deprivation therapy; HR, hazard ratio; CI, confidence interval; N/A, not avaliable

**Table 4:** The BCR/CP numbers were divided by the BED levels and ADT duration

BED level (Gy2)	<178.0				≥178.0			
	Total	BCR	%		Total	BCR	%	
No. (%)	146	13	8.9	8.9%	146	17	11.6	11.6%
No ADT use	74	11	14.9	*P *= 0.04	68	10	14.7	*P *> 0.05
ADT for 1–3 months	24	1	4.2	HR* *= 0.36	46	6	13.0	HR* *= 0.03
ADT for 4–12 months	48	1	2.1	95% CI* *= 0.14–0.93	32	1	3.1	95% CI* *= 0.34–1.39

BED level (Gy2)	<178.0				≥178.0			
	Total	CP	%		Total	CP	%	
No. (%)	146	7	4.8		146	6	4.1	
No ADT use	74	7	9.5	*P *= 0.02	68	3	4.4	*P *> 0.05
ADT for 1–3 months	24	0	0.0	HR* *= N/A	46	3	6.5	HR* *= 0.08
ADT for 4–12 months	48	0	0.0	95% CI* *= N/A	32	0	0.0	95% CI* *= 0.25–2.36

BCR, biochemical recurrence; CP, clinical progression; ADT, androgen deprivation therapy; BED, biologically equivalent dose; HR, hazard ratio; CI, confidence interval; N/A, not available.

The sites of CP in 13 patients are shown in Table 2. Five patients experienced LP: three showed lymph node involvement, four developed bone metastasis, and three developed lung metastasis during follow‐up (with duplication). All the five patients were diagnosed with LP, after the detections of BCR.

**Figure 1:**
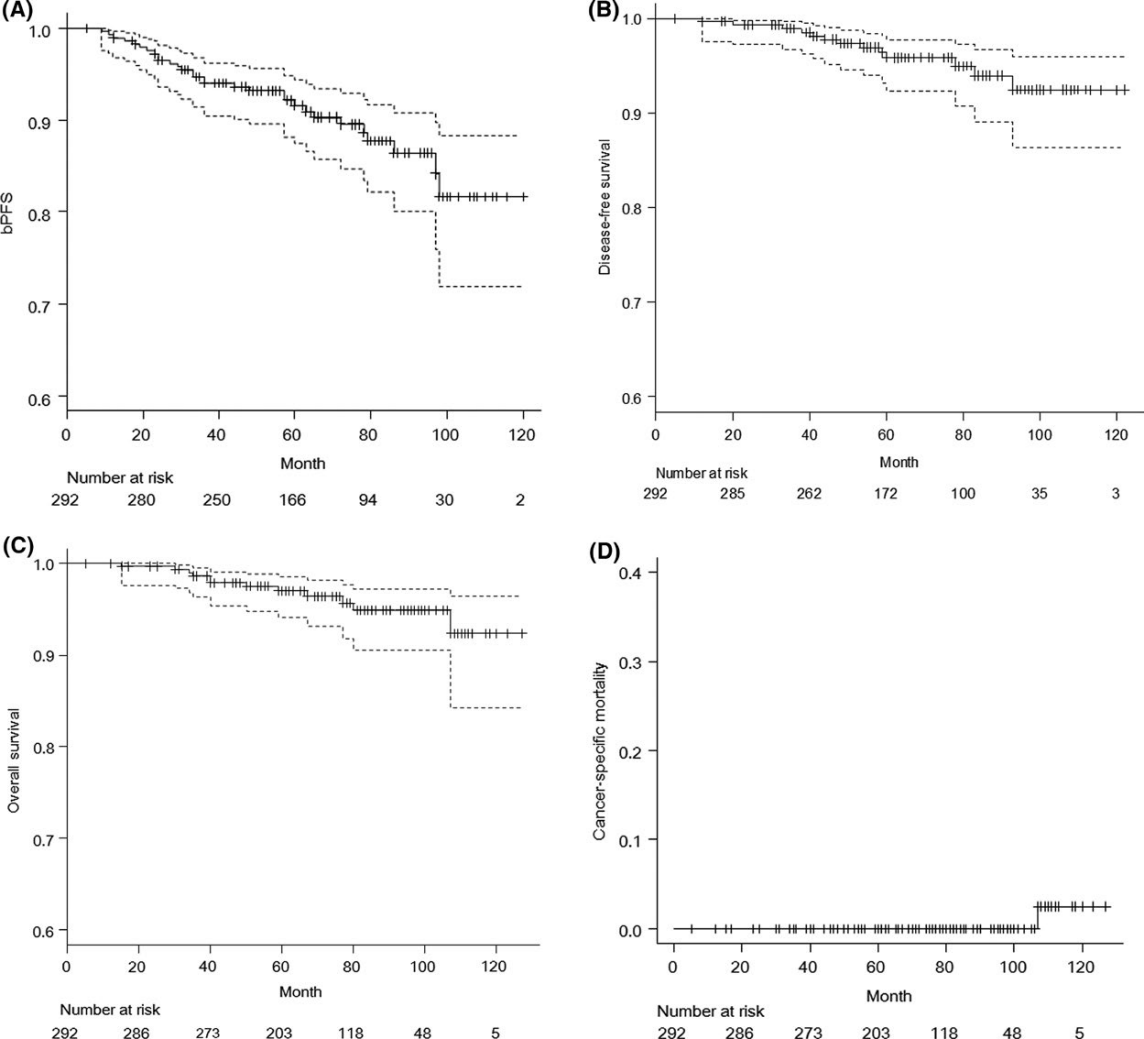
Kaplan–Meier curves for (A) biological progression‐free survival (bPFS) rates, (B) clinical progression‐free survival (cPFS), (C) overall survival (OS), and cumulative incidence analysis for (D) Cancer‐specific mortality in the entire cohort (*n *= 292).

**Figure 2:**
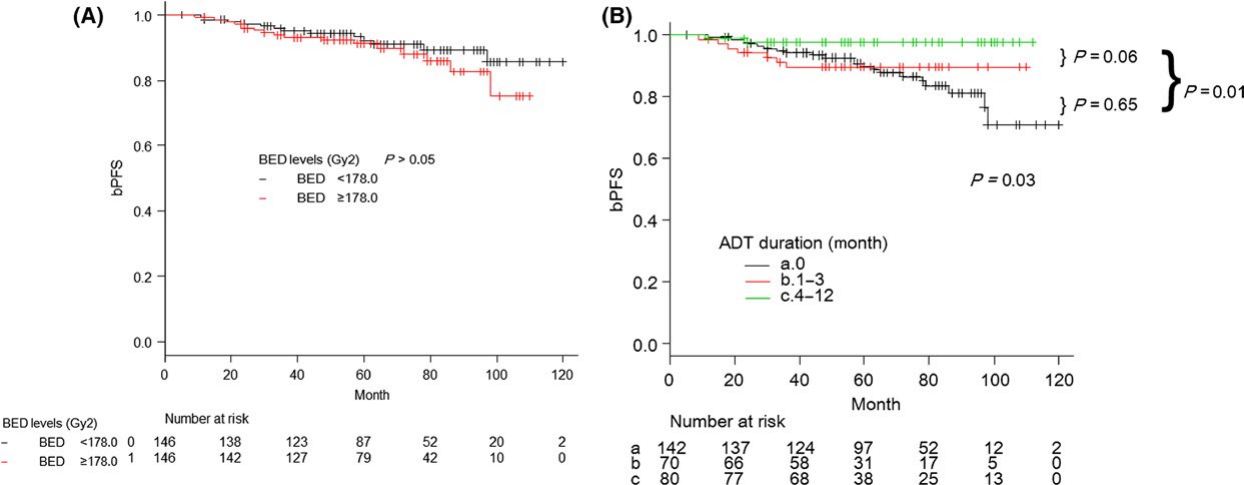
Kaplan–Meier curves for biological progression‐free survival (bPFS) rates based on (A) stratified biologically equivalent dose (BED) levels, and (B) divided androgen deprivation therapy (ADT) duration. Curves in the ADT subgroups had significant trend (*P *= 0.03).

The 5‐/7‐year bPFS rates were 91.6% (95% CI* *= 87.5–94.4%) and 87.7% (95% CI* *= 82.2–91.7%), respectively (Fig. 1A). The 5‐/7‐year cPFS rates for all patients were 95.9% (95% CI = 92.4–97.8%) and 94.0% (95% CI: 89.1–96.7%) (Fig. 1B). The 5‐/7‐year OS rates for all patients were 97.0% (95% CI: 94.1–98.5%) and 94.8% (95% CI: 90.5–97.2%) (Fig. 1C). We recorded 12 deaths, including one patient who died of PCa. The remaining patients died from other causes, including other malignancies in five patients, angina pectoris in one patient, and unspecified causes of death in five patients. The 5‐/7‐year cancer‐specific mortality rates were 2.6% (95% CI: 0.0–7.4%) each (Fig. 1D). Figure 2A and B show the bPFS according to BED levels and ADT duration. The bPFS rates at 5‐/7‐years for the low‐, and high‐dose groups were 92.1/89.3% and 91.2/86.0% (*P* > 0.05). The 5‐/7‐year bPFS rates for the no, short‐ and long‐ADT groups were 89.8/83.5%, 89.7/89.7%, and 97.5/97.5%, respectively, which had significant different trends (*P *= 0.03).

Table 5 shows the univariate and multivariate analyses for the prediction of bPFS. For the univariate analysis, age, PPC, and the use and duration of ADT (both continuous and categorical) were significant predicting factors for BCR, whereas BED, the PSA level, GS, primary Gleason grade, T stage, and supplemental EBRT were not. Multivariate analyses, depending on different ADT variables (ADT use, duration of ADT [both continuous and categorical]), indicated that age (*P *= 0.03–0.04), PPC > 50% (*P *= 0.01) were significant prognostic factors for BCR, whereas the ADT use itself was not a significant prognostic factor. Longer ADT duration (both continuous and categorical) was significantly associated with better bPFS (*P *= 0.04 and *P *= 0.02). In Table 6 showing the univariate analysis for cPFS, there were no variables indicating statistical significance despite some trend approaching significance in ADT duration (categorical, *P *= 0.053).

**Table 5:** Univariate and Multivariate (depending on the status of ADT covariant) analyses affecting bPFS

Covariant	Univariate			Covariant	Multivariate		
	*P* value	HR	95% CI		*P* value	HR	95% CI
Age year (continuous)	0.04	0.93	0.87–0.99	ADT use: yes vs. no	>0.05	0.47	0.21–1.03
BED (Gy_2_) (continuous)	>0.05	1.01	0.99–1.03	Age year (continuous)	0.03	0.93	0.86–0.99
BED (Gy_2_) (categorical)				PPC: <50% vs. ≥50%	0.01	2.47	1.20–5.08
<178.0 vs. ≥178.0	>0.05	1.43	0.69–2.96				
PSA: <10 vs. ≥10 (ng/mL)	>0.05	1.00	0.91–1.10				
GS:6 vs. 7	>0.05	4.53	0.61–33.4	ADT duration (continuous)	0.04	0.86	0.74–0.99
Primary Gleason grade: 3 vs. 4	>0.05	0.95	0.40–2.21	Age year (continuous)	0.04	0.93	0.87–0.99
T stage: T1 vs. T2	>0.05	1.73	0.77–3.92	PPC: <50% vs. ≥50%	0.01	2.53	1.23–5.19
PPC: <50% vs. ≥50%	0.01	2.46	1.20–5.04				
ADT use: yes vs. no	0.04	0.45	0.21–0.99				
ADT duration (continuous)	0.03	0.85	0.74–0.99	ADT duration (categorical)			
ADT duration (categorical)				0, 1–3, and 4–12 months	0.02	0.53	0.31–0.91
0, 1–3, and 4–12 months	0.01	0.52	0.30–0.88	Age year (continuous)	0.04	0.93	0.87–0.99
EBRT: yes vs. no	>0.05	0.42	0.13–1.38	PPC: <50% vs. ≥50%	0.01	2.47	1.20–5.07

bPFS, biological progression free survival; BED, biologically equivalent dose; PSA, prostate‐specific antigen; GS, Gleason score; PPC, percent positive biopsy core rate; ADT, androgen deprivation therapy; EBRT, external beam radiotherapy; HR, hazard ratio; CI, confidence interval

**Table 6:** Univariate analysis affecting cPFS

Covariant	Univariate		
	*P* value	HR	95% CI
Age year (continuous)	>0.05	1.03	0.92–1.14
BED (Gy_2_) (continuous)	>0.05	1.01	0.98–1.04
BED (Gy_2_) (categorical) <178.0 vs. ≥178.0	>0.05	0.89	0.30–2.67
PSA: <10 vs. ≥10 (ng/mL)	>0.05	1.14	0.37–3.49
GS:6 vs. 7	>0.05	1.85	0.24–14.31
Primary Gleason grade: 3 vs. 4	>0.05	0.97	0.26–3.55
T stage: T1 vs. T2	0.048	3.14	1.01–9.73
PPC: <50% vs. ≥50%	>0.05	2.78	0.93–8.29
ADT use: yes vs. no	>0.05	0.33	0.09–1.22
ADT duration (continuous)	>0.05	0.76	0.55–1.05
ADT duration (categorical) 0, 1–3, and 4–12 months	0.053	0.37	0.13–1.01
EBRT: yes vs. no	>0.05	0.70	0.15–3.16

cPFS, clinical progression free‐survival; BED, biologically equivalent dose; PSA, prostate‐specific antigen; GS, Gleason score; PPC, percent positive biopsy core rate; ADT, androgen deprivation therapy; EBRT, external beam radiotherapy; HR, hazard ratio; CI, confidence interval.

### Toxicities

Table S1 shows the incidence of genitourinary (GU) and gastrointestinal (GI) toxicity in this study. Urinary retention occurred in 10 patients (3.4%), and these patients all needed temporal urethral catheterization. One patient with grade 3 hematuria required hyperbaric oxygen therapy. Four of five patients with grade 3 hematochezia required intervention, and three underwent laser cauterization with or without clipping hemostasis. One patient underwent hyperbaric oxygen therapy. The remaining patient was managed conservatively but required blood transfusion. GU and GI toxicities were not significantly associated with the BED levels (*P > *0.05, in Table S2).

Five patients developed myocardial infarction after treatment, and one of three patients treated with ADT for 12 months died 30 months after treatment. One patient with a history of cerebral infarction and ADT for 12 months, later experienced recurrence. No distinct association was evident between these events and ADT use (Table S3).

## Discussion

The Japanese Guidelines for Safety Control of Brachytherapy with Permanently Implanted Sealed Radiation Sources for Prostate Cancer patient release criteria instruct that the measured radiation dose rate should not exceed 1.8 mSv/h at a distance of 1 m from the patient or that the administered radionuclide activity must be under 1300 MBq 16. Because of this guideline, prostatic volume reduction with neoadjuvant ADT is a common practice if the tumor exceeds 40 cc at the time of diagnosis 16, 17. Some patients in this study were enrolled in SHIP0804 for intermediate‐risk PCa, in which 3‐month neoadjuvant and none or 9‐month adjuvant ADT were an integral part of the protocol treatment 11. LDR‐BT delivers a higher BED than EBRT, and 81 Gy of EBRT, which is generally considered a high dose, delivered in 1.8‐Gy fraction equals a BED of 153 Gy_2_ 13. This BED is much lower than that used for LDR‐BT in our study (median: 178.0 Gy_2_). To date, the efficacy of ADT and its optimal duration remains controversial in LDR‐BT for intermediate‐risk PCa 18, 19. Therefore, we focused on the intermediate‐risk PCa population to further evaluate the role of ADT and the impact of high‐dose irradiation.

Marshall et al. reported ADT (for a median of 6 months) with LDR‐BT improved bPFS in intermediate‐risk PCa, based on the outcomes of 2495 patients 20. This study also showed that the longer use of ADT improved the bPFS (*P *= 0.02–0.04; Table 5).

The significance of BED for successful outcomes in LDR‐BT was originally described by Stock et al. in 1998 21. In the updated study, they showed BED was a significant predictor of bPFS and cause‐specific survival. They also reported that a BED < 152 Gy_2_ was inadequate for intermediate‐ and high‐risk PCa, and that high‐grade cancer required higher doses (>220 Gy_2_) for eradication 22, 23. However, this study failed to show a dose–response relationship with the bPFS, owing to several reasons, such as the small sample size and short follow‐up. Moreover, the narrower Gaussian distribution of BED in our study (interquartile range: 165–192 Gy_2_, median ± standard deviation: 178.0 ± 21.5 Gy_2_) compared to that reported by Stone et al. (158–215 Gy_2_, 192.0 ± 42.2 Gy_2_ [estimated from interquartile range]) 23, 24 could have significantly reduced the power of detection (Fig. S1) 22. According to Stone et al., patients with intermediate‐risk disease benefit the most from the highest BED (>200 Gy_2_) 25. The median BED level of 178.0 Gy_2_ in our study was lower than that reported in other studies (200–204.1 Gy_2_) 18, 20, which may be suboptimal to benefit intermediate‐risk PCa 18. Ultimately the impact of dose escalation in LDR‐BT might differ biologically from EBRT owing to the longer interval of radiation delivery. To clarify the efficacy of dose escalation in LDR, further prospective investigations will be needed.

Potential advantages in dose escalation must be balanced with treatment‐associated morbidity. The majority of GU and GI toxicities in our study were well tolerated. Although the incidence of grade 2 or greater proctitis was 21.3% (62/292), which was higher than that given in other reports 26, 27, the majority of these symptoms were relieved spontaneously. The incidences of comorbidities possibly associated with ADT, such as cardiovascular events (CVD), were lower than those reported in western countries, irrespective of the more liberal use of ADT 28. In this study, five patients (1.7%) developed myocardial infarctions, three of whom were treated with ADT, and three patients (1.0%) had congestive heart failures, only one of whom received ADT (for 2 months). Thus, CVD events were not significantly associated with the use of ADT. The impact of ADT on cardiovascular morbidities may vary among ethnic groups because the rate of mortality from ischemic heart disease is significantly lower in Japan than in the USA 29.

The limitation of this study is inherent in its retrospective and nonrandomized nature and sample size. Disturbances in erectile function after treatment were not evaluated. An additional weakness of our study is that the proportion of extremely high BED dose groups was smaller than other studies. Furthermore, the duration of ADT was not standardized. Randomized controlled trials are necessary to elucidate the optimal levels of BED and duration of ADT in LDR‐BT for patients with intermediate‐risk PCa. We do not have the data of testosterone recovery in this analysis, which is needed to exclude the possibility related to ADT affecting bPFS. This bias may not be large since median time to normalization of testosterone has been reported to be 6 months in patients who received ADT less than 24months 30. There also was a trend of improved cPFS in those with longer ADT (*P *= 0.053). Admittedly, longer follow‐up is needed to correctly appreciate the association.

In conclusion, the longer use of ADT, age, and higher PPC are significant predictors for bPFS after high‐dose irradiation with LDR‐BT in intermediate‐risk PCa. This possibility in the use of ADT needs to be explored further in a prospective way since it was found to improve outcomes without an evident increase in CVD toxicity.

## Conflict of Interest

None declared.

## Supporting information


**Figure S1.** Comparison of the distribution of biologically equivalent dose (BED) between Jikei University Hospital and Mount Sinai Medical Center.

**Table S1.** Genitourinary (GU) and gastrointestinal (GI) toxicity according to CTCAE ver 4.0 (Total 292 patients).

**Table S2.** Correlations of genitourinary (GU)/gastrointestinal (GI) toxicity and BED levels.

**Table S3.** Comorbidities related to androgen deprivation therapy.
