# Supplementary figures and images for: Do androgen deprivation and the biologically equivalent dose matter in low‐dose‐rate brachytherapy for intermediate‐risk prostate cancer?
Source: Cancer Med. 2016 Jul 25;5(9):2314–22. doi: 10.1002/cam4.820 (PMC5055153; doi:10.1002/cam4.820)

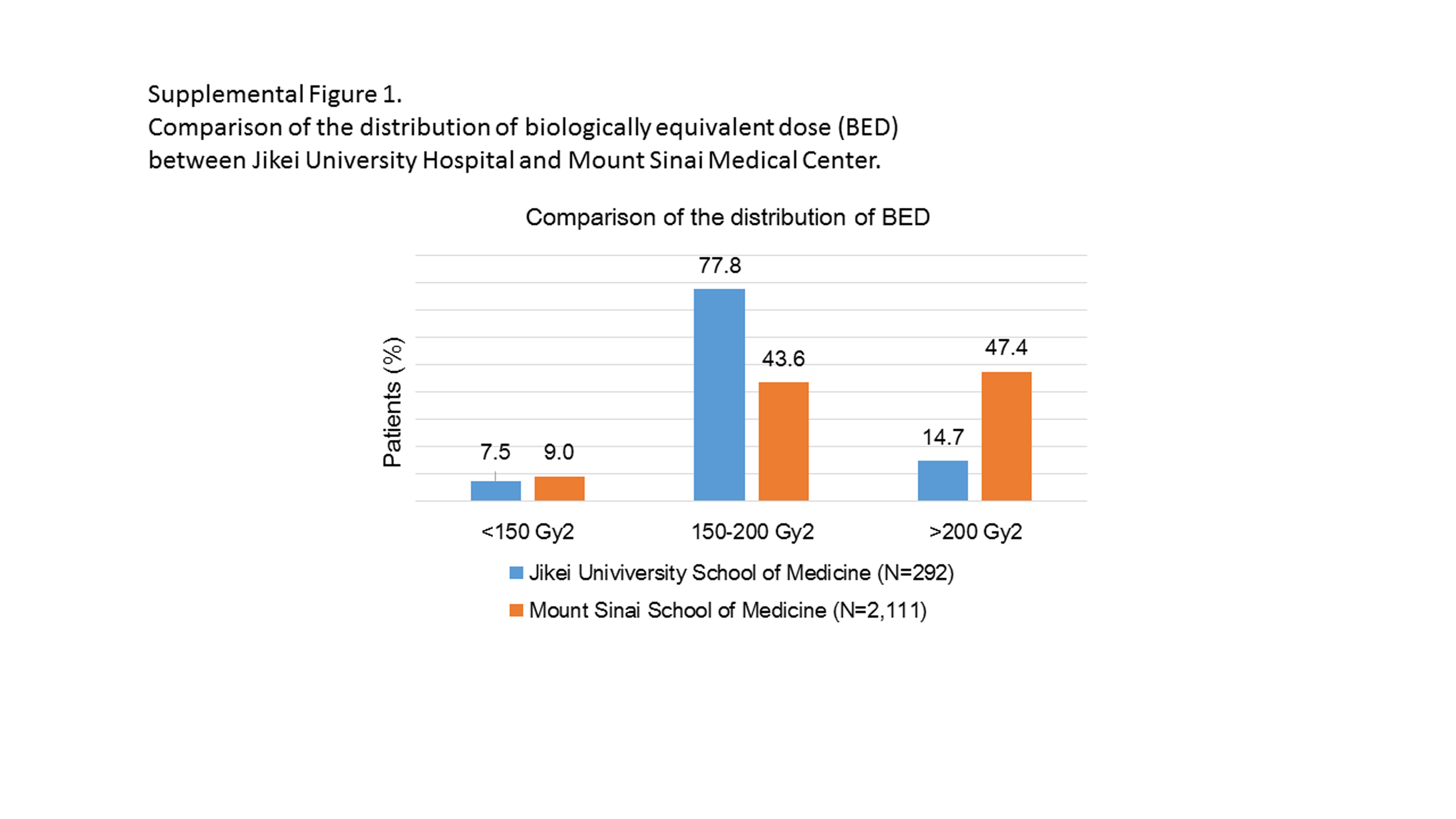

Supplement: Supplementary file 1 — Figure S1. Comparison of the distribution of biologically equivalent dose (BED) between Jikei University Hospital and Mount Sinai Medical Center. [file CAM4-5-2314-s001.tif]
