# Supplementary material for: Do androgen deprivation and the biologically equivalent dose matter in low‐dose‐rate brachytherapy for intermediate‐risk prostate cancer?
Source: Cancer Med. 2016 Jul 25;5(9):2314–22. doi: 10.1002/cam4.820 (PMC5055153; doi:10.1002/cam4.820)
Supplement: Supplementary file 2 — Table S1. Genitourinary (GU) and gastrointestinal (GI) toxicity according to CTCAE ver 4.0 (Total 292 patients). [file CAM4-5-2314-s002.docx]

| Supplemental Table 1 Genitourinary (GU) and gastrointestinal (GI) toxicity according to CTCAE ver 4.0 (Total 292 patients) | | | | | | | | |
| --- | --- | --- | --- | --- | --- | --- | --- | --- |
|  |  |  | Grade 2 |  | Grade 3 |  | Grade 4 |  |
|  |  |  | No. | % | No. | % | No. | % |
| Genitourinary (GU) | | Total ( without duplication) | 22 | 7.5 | 1 | 0.3 | 0 | 0 |
|  |  | Urinary frequency | 8 | 2.7 | ― | ― | ― | ― |
|  |  | Urinary incontinence | 3 | 1.0 | ― | ― | ― | ― |
|  |  | Urinary retention | 10 | 3.4 | 0 | 0 | 0 | 0 |
|  |  | Urinary urgency | 4 | 1.4 | ― | ― | ― | ― |
|  |  | Hematuria | 2 | 0.7 | 1 | 0.3 | 0 | 0 |
|  |  | Urinary tract pain | 1 | 0.3 | 0 | 0 | ― | ― |
|  |  |  | No. | % | No. | % | No. | % |
| Gastrointestinl (GI) | | Total ( without duplication) | 50 | 17.1 | 14 | 4.8 | 0 | 0 |
|  |  | Rectal pain | 0 | 0 | 0 | 0 | ― | ― |
|  |  | Hematochezia | 5 | 1.7 | 5 | 1.7 | 0 | 0 |
|  |  | Proctitis | 49 | 16.8 | 13 | 4.5 | 0 | 0 |
