# Supplementary material for: Do androgen deprivation and the biologically equivalent dose matter in low‐dose‐rate brachytherapy for intermediate‐risk prostate cancer?
Source: Cancer Med. 2016 Jul 25;5(9):2314–22. doi: 10.1002/cam4.820 (PMC5055153; doi:10.1002/cam4.820)
Supplement: Supplementary file 3 — Table S2. Correlations of genitourinary (GU)/gastrointestinal (GI) toxicity and BED levels. [file CAM4-5-2314-s003.docx]

| Supplemental Table 2 Correlations of genitourinary (GU) / gastrointestinal (GI) toxicity and BED levels | | | | | | |
| --- | --- | --- | --- | --- | --- | --- |
|  |  |  |  |  |  |  |
|  |  | BED level (Gy2) | | | | *P* value |
|  | No. | <178.0 | % | ≥178.0 | % |  |
| Total no. | 292 | 146 | 50.0 | 146 | 50.0 |  |
| GU≥ Grade 2 | 23 | 12 | 4.1 | 11 | 3.8 | >0.05 |
| GI≥Grade 2 | 64 | 29 | 9.9 | 35 | 12.0 | >0.05 |
| *Abbreviation*: BED = biologically equivalent dose | | | | | | |
