# Supplementary material for: Do androgen deprivation and the biologically equivalent dose matter in low‐dose‐rate brachytherapy for intermediate‐risk prostate cancer?
Source: Cancer Med. 2016 Jul 25;5(9):2314–22. doi: 10.1002/cam4.820 (PMC5055153; doi:10.1002/cam4.820)
Supplement: Supplementary file 4 — Table S3. Comorbidities related to androgen deprivation therapy. [file CAM4-5-2314-s004.docx]

| Supplemental Table 3 Comorbidities related to androgen deprivation therapy | | | | | | | | |
| --- | --- | --- | --- | --- | --- | --- | --- | --- |
| ADT duration (Month) | | | 0 | | 1-3 | | 4-12 | |
|  | No. | % | No. | % | No. | % | No. | % |
| Total no. | 292 | 100.0 | 142 | 100.0 | 70 | 100.0 | 80 | 100.0 |
| Comorbidity | 14 | 4.8 | 7 | 4.9 | 2 | 2.9 | 5 | 6.3 |
| Heart | 8 | 2.7 | 4 | 2.8 | 2 | 2.9 | 2 | 2.5 |
| Myocardial infarction (died) | 5 (1) | 1.7 (0.3) | 2 | 1.4 | 1 | 1.4 | 2 (1 died) | 2.5(1.3) |
| Congestive heart failure | 3 | 1.0 | 2 | 1.4 | 1 | 1.4 | 0 | 0.0 |
| Brain |  |  |  |  |  |  |  |  |
| Stroke | 6 | 2.1 | 3 | 2.1 | 0 | 0.0 | 3 | 3.8 |
| *Abbreviation*: ADT = androgen deprivation therapy | | | | | | | | |
